# Non-invasive Investigation of Tumor Metabolism and Acidosis by MRI-CEST Imaging

**DOI:** 10.3389/fonc.2020.00161

**Published:** 2020-02-18

**Authors:** Lorena Consolino, Annasofia Anemone, Martina Capozza, Antonella Carella, Pietro Irrera, Alessia Corrado, Chetan Dhakan, Martina Bracesco, Dario Livio Longo

**Affiliations:** ^1^Department of Nanomedicines and Theranostics, Institute for Experimental Molecular Imaging, RWTH Aachen University, Aachen, Germany; ^2^Department of Molecular Biotechnology and Health Sciences, Molecular Imaging Center, University of Torino, Turin, Italy; ^3^Institute of Biostructures and Bioimaging (IBB), Italian National Research Council (CNR), Turin, Italy; ^4^University of Campania “Luigi Vanvitelli”, Naples, Italy

**Keywords:** tumor metabolism, tumor acidosis, CEST-MRI, imaging, therapy, tumor pH

## Abstract

Altered metabolism is considered a core hallmark of cancer. By monitoring *in vivo* metabolites changes or characterizing the tumor microenvironment, non-invasive imaging approaches play a fundamental role in elucidating several aspects of tumor biology. Within the magnetic resonance imaging (MRI) modality, the chemical exchange saturation transfer (CEST) approach has emerged as a new technique that provides high spatial resolution and sensitivity for *in vivo* imaging of tumor metabolism and acidosis. This mini-review describes CEST-based methods to non-invasively investigate tumor metabolism and important metabolites involved, such as glucose and lactate, as well as measurement of tumor acidosis. Approaches that have been exploited to assess response to anticancer therapies will also be reported for each specific technique.

## Introduction

Outgrowing tumor mass typically displays an abnormal and disorganized vascular network, with poor functional vessels and extended hypoxic region (1, 2). Hypoxia is considered one of the major driving forces of tumorigenesis through the activation of the hypoxia-inducible factor 1 (HIF-1), that directly alters the expression of genes related to cell metabolism and proliferation (3). The induced metabolic modification markedly responds to tumor requirement for survival and expansion. On one side, the upregulation of the transmembrane receptor GLUT-1 ensures increased glucose avidity as a metabolic source of proliferation (4). On the other side, the metabolic switch to the glycolytic pathway exposes tumors to the paradoxically accumulation of acidic metabolites, as lactic acid and hydrogen ions, that results to be toxic for cancer cells. Therefore, the upregulation of dedicated proton transporters allows the extrusion of acidic products on the extracellular microenvironment, guarantees the maintenance of an aberrant pH gradient and induces the adaptation and clonal expansion of the most aggressive cells able to survive in such a hostile environment (5–7).

Considering the strategic role of metabolism on tumorigenesis, several targeting therapies have been developed to interfere with tumor expansion, alone or in combination with standard therapeutic treatments (8–13). Therefore, approaches for *in vivo* assessing the response to treatments and for improving tumor diagnosis are strongly required. In the clinical setting, positron-emission tomography (PET) technique is routinely exploited for measuring glucose uptake via 18F-fluorodeoxyglucose (FDG) injection, although radiation exposure limits repeated longitudinal studies (14–16). Furthermore, magnetic resonance imaging (MRI) offers a wide panel of approaches, by combining an optimal tissue contrast and good spatial information with acceptable sensitivity, to quantitatively interrogate several aspects of tumor microenvironment, including tumor metabolism and acidosis (17–20). One of the most promising and emerging technique for investigating tumor metabolism is the chemical exchange saturation transfer (CEST)-MRI (21, 22). CEST-MRI allows the detection of molecules endowed with mobile protons in chemical exchange with water. The application of radiofrequency (RF) pulses at specific offsets, corresponding to the absorbance peak of the mobile protons, nullifies the magnetization of the mobile protons, that become “saturated.” The exchange of the saturated protons with those of water molecules results in a transfer of reduced magnetization, hence in a decrease of the water signal, generating a (negative) contrast that can be detected by MRI. Consequently, many endogenous (proteins, peptides, sugars) or exogenous molecules owing exchangeable mobile protons can be imaged by CEST-MRI (23–25).

In this mini review, we will focus on CEST-MRI as a novel tool for imaging several aspects of tumor metabolism in both preclinical and clinical settings.

## Imaging Mobile Proteins (Amide Proton Transfer: APT)

Amide proton transfer (APT) imaging is a CEST-MRI approach that can detect the amide protons of endogenous mobile proteins and peptides that resonate at 3.5 ppm (26). APT imaging has been initially exploited for studies of ischemic stroke, neurologic disorders and brain tumors (27–32). Tumors exhibit a close relationship between unregulated proliferation and concentrations of mobile proteins, that may accumulate as defective products (33). Especially in high grade malignant brain tumors, the level of peptides and mobile proteins is substantially elevated (34). In Yan et al. the APT signal was compared between normal brain tissue and tumor in rats implanted with gliosarcoma. This study demonstrated that higher APT contrast in brain tumor correlated with an increased concentration of cytosolic proteins (35). In addition, APT imaging has been used for tumor characterization and diagnosis of brain tumors in patients (36–39). Furthermore, it is possible to use this innovative technique to differentiate between malignant gliomas and malignant lymphoma (40), to discriminate solitary brain metastases from glioblastoma (41) and to predict genetic mutations in gliomas, in particular the isocitrate dehydrogenase (IDH) mutation status (42, 43). Another feature that makes APT particularly interesting is its ability to differentiate between treatment-induced effects and true tumor progression (44, 45), providing a unique and non-invasive MRI biomarker for distinguishing viable malignancy from radiation necrosis and for predicting tumor response to therapy (46). In addition to brain tumors, APT imaging has been investigated in breast and prostate cancer. As it was demonstrated in brain tumors, APT imaging is able to discriminate between prostate cancer and non-cancer tissues, reporting an increase of cell proliferation rate and cellular density in tumor regions (47). Furthermore, variations in the APT signal have been observed in breast tumors, likely reporting about therapeutic effects and transformation of breast parenchyma (48, 49). In summary, APT imaging represents a promising biomarker for monitoring tumor progression and response to treatment and can be easily implemented in existing clinical scanners, despite further work is needed to remove confounding effects (protein concentration, pH, etc.) to the observed APT contrast (50–54).

## Imaging Glucose

Tumors typically display upregulated glucose uptake and glycolytic metabolism (55). In the clinical setting, PET imaging with the glucose analog FDG is considered the gold standard technique for non-invasively mapping glucose uptake and for assessing tumor response to conventional therapy (56). However, high maintenance costs and side effects related to radioactivity exposure of patients strongly limit the repeated applications of radionuclide techniques (57). Therefore, the idea of exploiting unlabeled D-glucose as an MRI contrast agent may represent a cheaper and potential alternative to FDG without involving ionizing radiations. Glucose molecules own five hydroxylic groups in fast exchange rate (500–6,000 Hz) with bulk water protons that can provide CEST contrast at 1–1.2 ppm from the water resonance (58, 59). The feasibility of imaging glucose uptake with the CEST-MRI technique was demonstrated in colorectal tumor xenograft murine models, with glucose contrast (GlucoCEST) correlated to FDG accumulation as measured by autoradiography (60). A different GlucoCEST contrast was also reported between two human breast tumor models characterized by different metabolic activity (58). In addition, the dynamic measurement of GlucoCEST contrast enhancements upon time (Dynamic Glucose Enhanced—DGE) following glucose injection showed increased penetration in brain tumors compared to the contralateral regions, demonstrating interesting application for brain tumors due to the reduced permeability of the blood brain barrier (61). One limitation of the GlucoCEST approach is the fast metabolism of native glucose that results in CEST contrast disappearance. Therefore, non-metabolizable glucose derivatives have been investigated for achieving prolonged contrast (=detectability) inside the tumor regions. Once phosphorylated by hexokinase enzymes, 2-Deoxy-D-glucose (2DG) remains entrapped in tumor cells and provides CEST contrast for long time, up to 90 min post injection (62, 63). However, the high doses required to generate enough contrast are not feasible for toxicity issues. A more promising molecule that has been intensively studied is the non-metabolizable 3-O-methyl-D-glucose (3OMG), that is considered non-toxic. Several studies tested 3OMG in different breast cancer models and showed higher uptake and CEST contrast in the more aggressive tumors, in according with the results obtained by FDG-PET (64–66). Beyond 3OMG, glucosamine (GlcN) and N-acetyl glucosamine (GlcNAc) can accumulate in tumors that overexpress the glucose transporters GLUT1 and GLUT2. These molecules were exploited as CEST contrast agents in breast and melanoma murine cancer models with different aggressiveness showing diverse accumulation inside the tumor (67, 68). Interesting results have been also obtained with low-calorie sweeteners, like sucralose, that was shown to provide CEST contrast in glioma tumor regions, and maltitol, that showed increased enhancement in brain tumors with compromised blood brain barrier (BBB) (69, 70).

**Figure 1:**
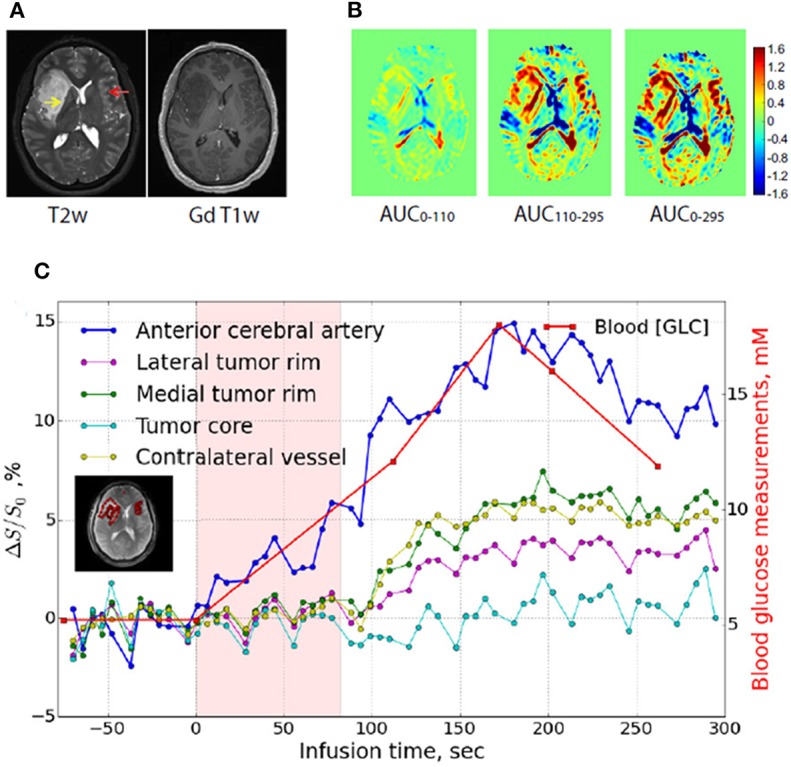
GlucoCEST imaging in human glioma tumor. **(A)** Anatomical (T2-weighted, left) and contrast-enhanced upon Gd-injection (T1-weighted, right) MR images in a glioma patient. **(B)** GlucoCEST contrast maps calculated as Area Under the Curve (AUC) showed at several time periods (0–110 s, left panel; 110–295 s, middle panel; 0295 s, right panel) indicate progressive accumulation of glucose inside tumor. **(C)** Dynamic glucoCEST contrast time curves for several brain regions (anterior cerebral artery, tumor core, lateral tumor rim, and contralateral vessel area). These curves show that glucose accumulation in lateral and medial tumor rim starts after 100 s of infusion, whereas the enhancement in the core area does not change over time. Reproduced with permission from Xu et al. (71).

Due to the high safety profile of glucose, its first use in patients was reported as early as 2015 in a glioma patients by using a high-field (7T) scanner (71). In comparison with the conventional small molecular weight Gd-based contrast agent, different areas of contrast enhancement were detected, suggesting that D-glucose may highlight tumor regions with different perfusion or permeability properties (Figures 1A,B). In addition, GlucoCEST contrast time curves highlighted potentially distinct biological areas of the brain tumor 10 min after D-glucose bolus infusion (Figures 1B,C). Another study investigated the GlucoCEST approach in head and neck cancer patients with a 3T scanner (72). Increased GlucoCEST contrast was registered in the tumor regions compared to muscle tissue and GlucoCEST enhancements were moderately correlated with FDG-PET results, despite a spatial mismatch likely reflecting the different metabolism between FDG and glucose. To improve the sensitivity of GlucoCEST, a similar approach that exploits the chemical exchange of mobile protons based on the Spin Lock method (dubbed CESL or chemical exchange spin lock) has been proposed for detecting glucose (73, 74). First results were obtained at high fields (9.4T) with a dynamic acquisition following glucose injection in glioma patients, demonstrating the feasibility of this approach for monitoring glucose accumulation in human brain tumors. Other studies showed a different glucose uptake in tumor brain regions in comparison to normal gray matter ones at lower magnetic fields (75), thus demonstrating its translational application at clinical level (76).

Overall, these results suggest that GlucoCEST could represent a valid alternative to FDG-PET for tumor diagnosis and staging, still several limitations, including reduced detectability at low field and origin of the glucose-based contrast arising from different compartments need to be tackled in the next years (77).

## Imaging Tumor Acidosis

### Intracellular Tumor pH Imaging

The amine and amide concentration-independent detection (AACID) approach is a recently developed CEST contrast mechanism that has been shown to be sensitive to intracellular pH changes (pHi). AACID CEST technique uses the ratio of the CEST effects generated by amide (Δω = 3.50 ppm) and amine (Δω = 2.75 ppm) protons from endogenous tissue proteins, which are predominantly from the intracellular space, for removing the concentration dependence. As a consequence, the measured CEST effect is only pH dependent, allowing to measure tumor intracellular pH (pHi) (78). McVicar et al. exploited the AACID CEST technique in a glioblastoma murine model to detect the selective acidification and decrease of pHi following the treatment with lonidamide, an anticancer drug that inhibits the monocarboxylic transporters (78). Similar results were obtained in glioblastoma murine models upon the administration of several pH-modulators such as topiramate, dichloroacetate and cariporide (79–82).

Another non-invasive pH-weighted imaging technique is the amine CEST approach, in which the amine protons (resonating at 3 ppm) of glutamine or glutamate molecules provide a pH-dependent (but not concentration independent) CEST contrast for mapping acidic tumor regions. Harris et al. applied this approach in both glioma murine models and in glioblastoma patients to detect acidic tumors and response to bevacizumab treatment (83, 84). Although the high translational potential of these endogenous approaches, concerns related to their capability to distinguish between intra- and extracellular pH contribution are still under consideration. In addition, variation of amide protons concentrations might be responsible of confounding effects resulting in less reliable pH estimations.

A recent approach to uncouple the contribution of concentration and exchange rate to the measured CEST contrast is that based on the omega-plot technique, initially developed to assess chemical exchange rates in paramagnetic contrast agents (85). Such approach has been improved and exploited for diamagnetic molecules *in vitro* (simulating complex endogenous systems) by simultaneous determination of labile proton ratio and exchange rate (that is dependent on pH) (86, 87). Although not yet demonstrated, the omega plot approach may provide useful information for intracellular pH, but further technical advancements are needed to translate it *in vivo*.

### Extracellular Tumor pH Imaging

**Figure 2:**
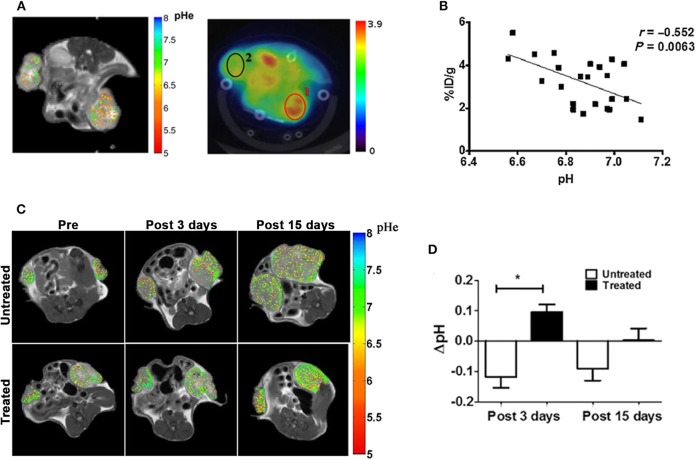
MRI-CEST tumor pH imaging upon iopamidol injection in murine tumors. **(A)** CEST-MRI pHe maps of a breast cancer tumor overlaid on anatomical MRI image upon iopamidol injection (left) and FDG-PET image overlaid on CT image (middle) upon FDG injection in the same mouse. The tumor on the right side shows lower pHe values in the MRI-CEST pHe map corresponding to higher FDG uptake in the PET image. **(B)** Correlation plot between FDG-PET uptake and tumor pHe values shows a significant inverse correlation between FDG uptake (% ID/g) and tumor pH values. Reproduced with permission from Longo et al. (96). **(C)** Representative CEST-MRI tumor pHe maps overimposed on anatomical reference images before, 3 and 15 days after treatment with dichloroacetate for treated and untreated mice. Images show increased number of less acidic pixel in treated tumors upon dichloracetate therapy in comparison to control mates. **(D)** Bar graphs show a significant reduction in tumor acidosis after 3 days of treatment in treated tumors compared to untreated mates, whereas a restoration of tumor acidosis, likely reflecting the onset of tumor resistance is reported after 15 days of treatment (*P < 0.05, Student's *t* test). Reproduced with permission from Anemone et al. (97).

To overcome the limitations of endogenous CEST-MRI techniques, exogenous molecules have been exploited as extracellular tumor pH reporters for CEST-MRI applications. In the last decade, great expectations surrounded the class of the X-ray FDA-approved iodinated contrast media, considering their high safety profile and translational potential (88). Due to their hydrophilic chemical structure, iodinated agents remain confined outside the cells and can be visualized as perfusion agents in tumor by CEST-MRI (89, 90). Their first application as pH CEST-MRI agents involved the use of iopamidol (Isovue®, Bracco Diagnostic), possessing two amide proton pools that can be saturated at 4.2 and 5.5 ppm (91, 92). The set-up of a ratiometric procedure allows to accurately measure extracellular tissue pH (pHe) in the pH range of 5.5–7.9, independently of the contrast agent concentration, with an accuracy of 0.1 units at several magnetic fields (93–95). CEST-MRI tumor pH imaging was combined to FDG-PET to elucidate the deregulation of tumor metabolism in a breast cancer model (96). This work evidenced that tumor regions with more acidic pHe show increased FDG uptake and demonstrated *in vivo*, for the first time, the relationship between tumor acidosis and high glycolytic rate. In addition, it provided evidence of the feasibility of measuring tumor pH heterogeneity at the clinical field of 3T (Figures 2A,B). The combination of CEST pH-imaging and FDG-PET was then exploited for predicting the early therapeutic efficacy of metformin in a preclinical model of pancreatic cancer (98). In addition, the possibility to measure tumor pHe opened new routes for monitoring the effect of novel anticancer treatments that can reverse the glycolytic tumor phenotype (97). Anemone et al. showed that this approach can monitor early pH changes in a breast murine cancer model upon the treatment with dichloroacetate, a small compound targeting mitochondria, and that can be exploited to detect the onset of the resistance, hence providing useful insights about the therapeutic efficacy (Figures 2C,D).

Another iodinated agent used for pH mapping is iopromide (Ultravist®, Bayer Healthcare), that has two amide pools resonating at 4.6 and 5.6 ppm that can be exploited to measure tumor pH within the 6.5–7.2 range (99). CEST-MRI with iopromide revealed that breast cancer models with different histopathological features show significant differences in pHe values and that tumor acidosis is associated with metabolic biomarkers in B-lymphoma xenografts (100, 101). In addition, a comparative study between iopromide and iopamidol showed that although these agents measured similar pH values *in vivo*, iopamidol reveals more accurate pH measurement (102).

One of the main advantages of this class of agents relies in their very high safety profile for administration in patients. Consequently, CEST-MRI pH imaging with iopamidol was initially translated for measuring kidney and bladder pH in healthy volunteers (103–105). Later on, the capability to provide accurate tumor pH maps was demonstrated with iopamidol in both breast and ovarian cancer patients showing acidic tumor pH values (106). These preliminary results pointed out that efficient translation still requires optimization of several aspects, including acquisition protocol and data analysis to further evaluate the diagnostic and therapeutic utility of tumor pH mapping in the clinical setting. To this purpose, different studies aimed to optimize RF irradiation, reduce respiration artifacts and enlarge the body coverage acquisition have been performed (107–109). In addition, new ratiometric approaches have been formulated to extend the use of iodinated agents even with a single resonating protons for pH measurements (110, 111). Promising results have been obtained with iobitridol (Xenetix®, Guerbet), showing accurate pH measurement in murine tumors once irradiated with different power levels (112).

PARACEST pH-responsive agents are characterized by a large chemical shift of the mobile protons from the water peak that should improve their detectability in comparison to DIACEST molecules, as iodinated agents or glucose (23, 113). The Yb-HPDO3A contrast agent has been exploited for measuring tumor pHe in both melanoma and in glioma murine models (114, 115). Interestingly, in the melanoma model changes in tumor pHe were observed and correlated with the tumor progression stage. Similar approaches based on other PARACEST agents allowed to measure tumor pHe in rat brain tumor models, although direct injection of the contrast agent in the tumor and renal ligation were needed to maintain high concentrations of the agent for measuring pH (116–118). Currently, the high saturation power needed to generate enough CEST contrast limits a wider applicability of these pH responsive PARACEST agents, however molecules with optimal exchange rates have been recently proposed (119).

## Imaging Lactate

The preferential ATP production via glycolysis of glucose to lactate leads to high lactate levels that some cancer cells can even exploit as a metabolic fuel (120–122). Conventionally, lactate can be observed and quantified by Magnetic Resonance Spectroscopy (MRS) or by the recently developed hyperpolarization technique (123–129). However, these methods are limited by low spatial resolution and long acquisition times. The chemical shift of the hydroxylic proton of the lactate is very close to the water signal and renders quite difficult to directly detect lactate *in vivo* by CEST imaging. However, correlation of the signal arising from lactate between CEST and MR spectroscopy has been performed in a lymphoma murine tumor upon lactate infusion (130) or in a mitochondrial disease model (131). Other approaches exploited lactate-responsive PARACEST contrast agents for taking advantage of the larger chemical shift difference of these molecules and the CEST contrast dependence with lactate concentration (132, 133). Zhang et al. (134) demonstrated the feasibility of this approach by measuring lactate excreted from lung cancer cells in tissue culture.

## Conclusion and Future Perspectives

In summary, CEST-MRI imaging is a fast-expanding field with enormous potential to assess several aspects of tumor metabolism. Moreover, since tumor acidosis is a general feature in all tumors, imaging tumor pH might become a powerful and wide tool for oncological imaging at both preclinical and clinical level. First studies in patients demonstrated the feasibility of these novel imaging approaches for imaging human tumors. Further improvements in fast acquisition sequences, post-processing and standardization set-up are mandatory for the widespread use of CEST-MRI in the clinical settings. Despite the fundamental insights that imaging tumor acidosis with iopamidol can provide, additional studies are needed to validate it in comparison to established clinical approaches and to demonstrate that it can be exploited for monitoring treatment response to (novel) anticancer therapies.

## Author Contributions

DL and LC conceived, structured, and edited the mini review article. DL, LC, AA, MC, ACa, PI, CD, ACo, and MB each wrote individual sections of the mini review article and critically revised it for intellectual content. All authors provided final approval of the version of the article submitted for publication.

### Conflict of Interest

The authors declare that the research was conducted in the absence of any commercial or financial relationships that could be construed as a potential conflict of interest.
